# Towards Engineering Hormone-Binding Globulins as Drug Delivery Agents

**DOI:** 10.1371/journal.pone.0113402

**Published:** 2014-11-26

**Authors:** Wee Lee Chan, Aiwu Zhou, Randy J. Read

**Affiliations:** 1 Department of Haematology, University of Cambridge, Cambridge Institute for Medical Research, Addenbrooke's Hospital, Cambridge, United Kingdom; 2 Key Laboratory of Cell Differentiation and Apoptosis of Ministry of Education of China, Shanghai Jiao Tong University, School of Medicine, Shanghai, People's Republic of China; Monash University, Australia

## Abstract

The treatment of many diseases such as cancer requires the use of drugs that can cause severe side effects. Off-target toxicity can often be reduced simply by directing the drugs specifically to sites of diseases. Amidst increasingly sophisticated methods of targeted drug delivery, we observed that Nature has already evolved elegant means of sending biological molecules to where they are needed. One such example is corticosteroid binding globulin (CBG), the major carrier of the anti-inflammatory hormone, cortisol. Targeted release of cortisol is triggered by cleavage of CBG's reactive centre loop by elastase, a protease released by neutrophils in inflamed tissues. This work aimed to establish the feasibility of exploiting this mechanism to carry therapeutic agents to defined locations. The reactive centre loop of CBG was altered with site-directed mutagenesis to favour cleavage by other proteases, to alter the sites at which it would release its cargo. Mutagenesis succeeded in making CBG a substrate for either prostate specific antigen (PSA), a prostate-specific serine protease, or thrombin, a key protease in the blood coagulation cascade. PSA is conspicuously overproduced in prostatic hyperplasia and is, therefore, a good way of targeting hyperplastic prostate tissues. Thrombin is released during clotting and consequently is ideal for conferring specificity to thrombotic sites. Using fluorescence-based titration assays, we also showed that CBG can be engineered to bind a new compound, thyroxine-6-carboxyfluorescein, instead of its physiological ligand, cortisol, thereby demonstrating that it is possible to tailor the hormone binding site to deliver a therapeutic drug. In addition, we proved that the efficiency with which CBG releases bound ligand can be increased by introducing some well-placed mutations. This proof-of-concept study has raised the prospect of a novel means of targeted drug delivery, using the serpin conformational change to combat the problem of off-target effects in the treatment of diseases.

## Introduction

Since antiquity, Man has sought to use compounds extracted from plants to treat diseases such as cancer, often with little success. It was not until the interbellum years that the modern era of cancer chemotherapy really took hold, when toxic chemicals were found to be effective against this hitherto incurable disease. Nearly seventy years after the first real effective drug against cancer, nitrogen mustard (Bis(2-choroethyl)methylamine hydrochloride), was first introduced into clinical use [1], the vast majority of anti-cancer drugs are still highly toxic. These drugs need to kill tumour cells using overwhelming cytotoxicity while remaining innocuous to healthy tissue. Unfortunately, with little to differentiate between the two, it has frequently been found that the benefits in terms of overall survival are often marginal, while the risks of off-target toxicity, whether lethal or chronic, are high [2]. As a result, chemotherapy often causes a plethora of side-effects ranging from emesis, stomatitis and alopecia that reduce the patient's quality of life, to leukopenia, febrile neutropenia and sepsis that are potentially debilitating [3], [4].

To reduce the incidence of these off-target toxicities, chemotherapeutic agents need to be targeted specifically to tumours. One of the earliest breakthroughs in attaining site-specificity in cancer therapy was the formulation of a styrene-maleic acid copolymer-conjugated neocarzinostatin (SMANCS), where the potent anti-tumour agent, neocarzinostatin, was attached to a lipophilic polymer, allowing it to penetrate more efficiently into solid tumours than intramuscular injection of the non-derivatised drug [5]–[7]. However, SMANCS and other similar polymer conjugates were found to be limited in their effectiveness in cases involving hypovascular tumours such as those seen in prostatic and pancreatic cancers [8]. Since then, a new class of antibody-based anti-cancer drugs has emerged. Some of these comprise antibodies conjugated to therapeutic drugs. The antibody recognises certain antigens specific to the tumour tissue, thus selectively directing the drug to the target. ‘Naked' antibodies have also been used to target tumour tissues directly for destruction by the body's immune system. There are currently three antibody-drug conjugates and 11 ‘naked’ monoclonal antibodies approved by the Food and Drug Administration for the treatment of cancers [9], [10]. Another form of immunotherapy that has gained traction in recent years is therapeutic vaccination. One such vaccine, Sipuleucel-T, has recently been approved for use on prostate cancer. This involves extracting the patient's antigen-presenting cells and activating them in vitro with the tumour-associated antigen, prostate acid phosphatase, before injecting them back into the patient to prime a T cell response against the tumour [11], [12].

Despite numerous advances in the field, many of these new targeting methods are still not sufficiently specific to prevent off-target effects. Lipophilic conjugates were found to accumulate in neovasculature regardless of the tissue type [8], while many of the antibody conjugates and antibodies developed for cancer therapy target entire classes of proteins such as epithelial growth factor receptors (EGFRs) and kinases, which are found in both tumour and healthy tissues. As a consequence, some drugs such as cetuximab and gefitinib can cause serious side effects [4]. Therapeutic vaccines appear to be fairly specific, but as they need to be tailored specifically for the individual patient, they are extremely costly, with a full course of Sipuleucel-T treatment currently priced at $93,000 [13].

While ever more sophisticated means of delivering medicine to specific locations in the body are being developed to overcome this problem of non-specificity, we observed that Nature has already evolved elegant ways of sending biological molecules to where they are needed. The hormone binding globulins, corticosteroid binding globulin (CBG) and thyroxine binding globulin (TBG), are examples of naturally occurring site-specific carriers. These hormone carriers release their respective ligands, cortisol in the case of CBG and thyroxine in the case of TBG, when their reactive centre loops are cleaved by elastase, a protease released by neutrophils at sites of inflammation [14], taking advantage of the site and temporal specificity dictated by neutrophil elastase secretion. Despite not possessing any inhibitory activity, CBG and TBG belong to a superfamily of proteins known as serine protease inhibitors (SERPINS) [15], [16]. Upon cleavage of their reactive centre loops, both hormone binding globulins undergo the canonical serpin conformational change that involves the incorporation of the cleaved reactive centre loop into the central β-sheet A as a new β-strand. This pronounced conformational change is the basis for the inhibitory mechanism of inhibitory serpins [17], and in CBG and TBG gives rise to a change in ligand binding affinity, allowing the release of hormones at inflammatory loci [18]–[20].

In this work, we used CBG as a case study. We report that we have successfully proved the concept that hormone binding globulins can, in principle, be engineered to carry a non-physiological ligand to defined locations in the body. By optimising the various physical characteristics of the engineered protein in the future, it could potentially be employed as a drug delivery agent in cancer chemotherapy.

## Materials and Methods

### Recombinant CBG

Wild type and engineered human CBG were expressed in the BL21star (DE3) strain of Escherichia coli using the pSUMO3 expression system and purified from the bacterial lysate using fast protein liquid chromatography, as previously described [21]. Purified samples of the protein were stored as 1 mg/ml solutions in 10 mM Tris-HCl, pH 7.4, 150 mM NaCl, 1 mM EDTA at −80°C until they were used.

### Fluorimetric determination of binding affinity

CBG is known to bind cortisol with a one-to-one stoichiometry [18], [19]. Therefore, the dissociation constant, which is a measure of binding affinity, is given by the following equation:
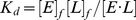
(1) where [E]_f_ is the concentration of free CBG, [L]_f_ is the concentration of free ligand, and [E·L] is the concentration of the CBG-ligand complex.

Fluorescence spectroscopy was used to study the CBG-ligand interactions. Experiments were performed on an LS55 120V fluorescence spectrometer (Perkin Elmer), and the data were read and recorded using their proprietary FL WinLab software. For binding studies of CBG with cortisol, the excitation wavelength was set at 280 nm and the emission was detected at 350 nm, with a 315 nm cut-off filter used to prevent the excitation laser from leaching into the emission channel. For the binding studies of engineered CBG with L-T4-thyroxine-6-carboxyfluorescein, the excitation wavelength was set at 495 nm and emission was recorded at 520 nm whilst applying a 515 nm cut-off filter.

The cortisol stock solution was prepared by dissolving lyophilised cortisol (Sigma Aldrich) in 80% ethanol to make a 500 µM solution, which was then diluted with water to make solutions of 20, 40 and 80 µM, with water being used as a negative control. Aliquots of the ligand solutions were then titrated into 800 µl of a 200 nM solution of CBG made by diluting the protein stock with a buffer containing 10 mM Tris-HCl, pH 7.4, 150 mM NaCl, 1 mM EDTA, 0.1% v/v PEG 8000, and the decrease in fluorescence due to quenching was measured.

L-T4-thyroxine-6-carboxyfluorescein was previously synthesised and purified as a 1.09 µM solution with acetonitrile as its solvent [20]. This was diluted into a 5 nM solution with a buffer containing 10 mM Tris-HCl, pH 7.4, 150 mM NaCl, 1 mM EDTA, 0.1% v/v PEG 8000. Aliquots of the engineered CBG were titrated into 800 µl of this solution, and increments in fluorescence signal were monitored.

In both cases, the data were fitted using Prism 5 (GraphPad Software) to the following equation to obtain the dissociation constant, K_d_: 
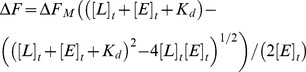
(2) where ΔF is the fluorescence change, ΔF_M_ is the maximum change in fluorescence signal, [L]_t_ is the total concentration of ligand added and [E]_t_ is the total protein concentration.

### Biolayer interferometry

Biolayer interferometry was carried out on a ForteBio Octet Red apparatus (ForteBio, Menlo Park, California, USA) to determine binding affinity where fluorescence quenching experiments would not work due to mutation of Trp 371, which is the main source of intrinsic fluorescence in the binding pocket. The system uses a change in interference pattern of reflected light to detect the change in optical thickness of the sensor tip. Cortisol was immobilised on the biosensor tip surface by forming an adduct of hydrocortisone 21-hemisuccinate and a pentylamine-biotin linker through an amide bond, then allowing the biotin moiety on this cortisol adduct to bind to the streptavidin-conjugated biosensor tip. The cortisol-conjugated sensors were immersed into wells of a shaking microtitre plate containing solutions of CBG at 0, 200, 500, 1000, 2500, 5000 and 10000 nM concentrations in a 10 mM Tris-HCl, pH 7.4, 150 mM NaCl, 1 mM EDTA, 0.1 mg/ml bovine serum albumin buffer, and analysed against an internal reference.

### Protease cleavage assay

To determine if a particular target protease is able to cleave the reactive centre loop of an engineered CBG variant, the protease was incubated with CBG at a molar ratio of 1∶100 at 37°C for half an hour unless otherwise specified. Reactive loop cleavage is marked by a characteristic 4 kDa change in molecular weight following release of a peptide when the R-state CBG is unfolded, such as when it is subjected to the denaturing conditions of an SDS-PAGE [14], [22]. We used this as a convenient means by which to check the engineered protein's protease specificity and, as a corollary, its site specificity.

### Crystallisation and data collection

The structure of CBG-S100C-V236C-T349R was determined experimentally by X-ray crystallography. The protein was expressed and purified from E.coli. It was then concentrated to 5 mg/ml in 10 mM Tris-HCl, pH 7.4, 150 mM NaCl, 1 mM EDTA and used to set up sitting drops containing 2 µl protein and 2 µl reservoir solution (12% PEG 3350, 50 mM MES, pH 5.3, 200 mM NaCl). Initial attempts gave no crystals, but cross-seeding the drops with fragments of crystals of reactive loop-cleaved chimeric CBG-AAT yielded crystal clusters after incubating for three days at 20°C. Single CBG-S100C-V236C-T349R crystals were eventually obtained by macroseeding sitting drops containing 2 µl protein and 2 µl reservoir solution (18% PEG 3350, 50 mM MES, pH 5.3, 200 mM NaCl) with fragments of crystals from the clusters. It is worth noting that the initial seed crystals were a different crystal form from the resultant CBG-S100C-V236C-T349R crystals. The CBG-AAT seed crystals had P1 crystallographic symmetry, while CBG-S100C-V236C-T349R crystallised in space group P2_1_2_1_2_1_.

The crystals were soaked in a cryoprotectant (12% PEG 3350, 50 mM MES, pH 5.3, 200 mM NaCl, 20% ethylene glycol) and then cryocooled in liquid nitrogen. A 1.8 Å data set was collected at station I03 of the Diamond Light Source. The data were processed with Mosflm [23] and scaled with Aimless [24]. The structure was then solved by molecular replacement with Phaser [25], using the coordinates of cleaved CBG-AAT chimera (PDB 2VDY [19]) as the search model. Prior to molecular replacement, the search model was first prepared using Sculptor [26] to downweight unreliable and non-identical parts of the structure. The atomic model was then rebuilt in Coot [27] and refined using Phenix. refine [28]. One copy of the protein was found in the asymmetric unit, and no external model restraints were used for refinement. The structure was refined with good geometry and validated using MolProbity [29]. Final refinement statistics are listed in Table 1. The atomic coordinates and structure factors have been deposited in the Protein Data Bank as entry 4C41. Diagrams of the models were generated using the open source molecular graphics software, PyMol (The PyMOL Molecular Graphics System, Version 1.3 Schrödinger LLC).

**Table 1:** Crystallographic statistics.

	PDB 4C41
	(cleaved CBG Ser100Cys-Val236Cys)
**Wavelength (Å)**	0.9763
**Resolution range (Å)**	40.0–1.80 (1.864–1.80)*
**Space group**	P2_1_2_1_2_1_
**Unit cell**	*a* = 42.13 *b* = 73.39 *c* = 126.69
**Total reflections**	249310 (16711)
**Unique reflections**	31823 (2165)
**Multiplicity**	7.9 (7.7)
**Completeness (%)**	86.0 (98.4)
**Mean I/sigma(I)**	8.4 (2.6)
**Wilson B-factor (Å^2^)**	16.3
**R-merge**	0.148 (0.810)
**R-meas**	0.160
**CC_1/2_**	0.995 (0.644)
CC*	0.999 (0.886)
**R-work**	0.176 (0.254)
**R-free**	0.206 (0.261)
**Number of atoms**	3115
**– macromolecules**	2820
**– solvent**	295
**Protein residues**	367
**RMS bonds (Å)**	0.003
**RMS angles (○)**	0.80
**Ramachandran favoured (%)**	98.9
**Ramachandran outliers (%)**	0.3
**Clashscore**	0.51
**Average B-factor (Å^2^)**	20.6
**– macromolecules**	19.3
**– solvent**	32.0

*high resolution shell in parentheses.

## Results and Discussion

### Altering site specificity

CBG is secreted in the “stressed” (S) conformation, in which the reactive centre loop is intact. In the body, cleavage of the reactive centre loop by neutrophil elastase causes it to undergo a conformational change to the “relaxed” (R) conformation, resulting in the release of its ligand, cortisol [14]. It is therefore evident that when and where CBG acts in the body is controlled to a large extent by the proteolytic cleavage of the protein's reactive centre loop. Consequently, the primary determinants of a hormone binding globulin's site specificity are the amino acid sequence of its reactive centre loop, which decides its target serine protease, and the localisation of this protease.

The reactive centre loop of CBG is a 17 residue region linking s3A of β-sheet A on its N-terminal end to s1C of β-sheet C on its C-terminal end [17], [30]. X-ray structures of protease-serpin complexes [31], [32] show that there are extensive interactions between the serpin's reactive centre loop from residue P4' to P4 (Schechter-Berger nomenclature [33]) and the binding pockets of the serine protease, suggesting that one or more of these residues are important for recognition by the protease.

#### Targeting sites of thrombosis

Thrombin, also known as Factor IIa, is a key component of the blood coagulation cascade. It is a serine protease, and its interaction with various substrates and cofactors in the coagulation network is vital to the maintenance of haemostasis. In the event of vascular injury, activation of the coagulation cascade causes the zymogen, prothrombin, to be cleaved to produce enzymatically-active thrombin, which then goes on to cleave fibrinogen to fibrin. Fibrin polymerises to form the beginning of a haemostatic plug [34]. The activity of thrombin is typically regulated via both the coagulation cascade and the action of antithrombin, a serpin that functions as a thrombin-specific suicide inhibitor. Dysregulation of the coagulation network, and consequently thrombin function, can result in a number of pathologies ranging from coagulopathic bleeding on one extreme to atherothrombotic disease on the other.

**Figure 1:**
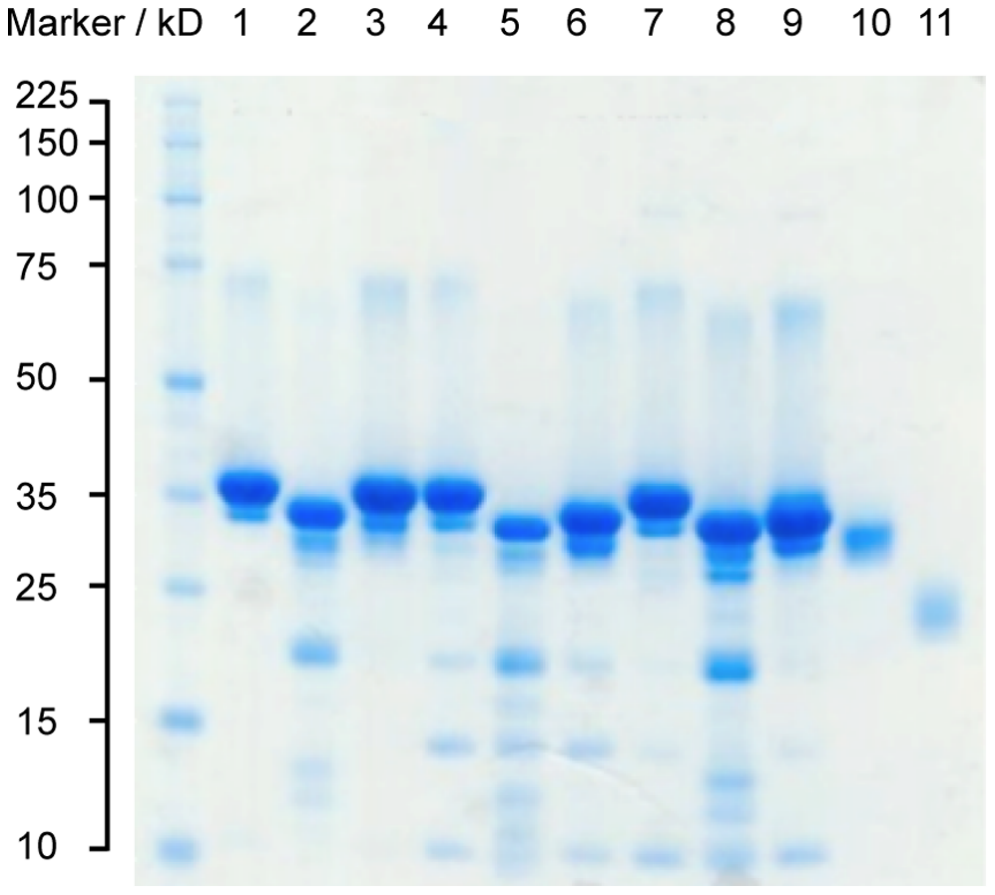
Thrombin specificity assay. Wild type and engineered CBG were incubated with thrombin. Only CBG-antitrypsin_Pittsburgh_ and CBG-Thr349Arg were successfully cleaved by thrombin, while wild type CBG remained unreactive. Lane 1: wild type CBG (negative control); Lane 2: wild type CBG + human neutrophil elastase; Lane 3: wild type CBG + thrombin; Lane 4: CBG-antitrypsin_Pittsburgh_ (negative control); Lane 5: CBG-antitrypsin_Pittsburgh_ + human neutrophil elastase; Lane 6: CBG-antitrypsin_Pittsburgh_ + thrombin; Lane 7: CBG-Thr349Arg (negative control); Lane 8: CBG-Thr349Arg + human neutrophil elastase; Lane 9: CBG-Thr349Arg + thrombin; Lane 10: thrombin; Lane 11: human neutrophil elastase. Lanes 5 and 8 show that further engineering would be required to remove elastase cleavage.

We have previously shown that by substituting the P15 to P1′ amino acid residues of wild type human CBG (GVDTAGSTGVTLNLTS) with those of the Pittsburgh variant of α_1_-antitrypsin (GTEAAGAMFLEAIPRS), we could make CBG susceptible to cleavage by thrombin [19]. This variant of α_1_-antitrypsin was first discovered in 1983 in a patient who died from an episodic bleeding disorder [35], and bore a methionine to arginine substitution in the P1 position of the reactive centre loop. This mutation alone turned α_1_-antitrypsin into the functional equivalent of antithrombin. Although exosites such as the heparin binding site in antithrombin contribute to protease recognition, this result suggested that the identity of the residue at the P1 position is the single most important factor for conferring specificity in serpins, and that changing the P1 residue to arginine can be sufficient to make a serpin susceptible to proteolysis by thrombin. Therefore, we made a single point mutation in wild type CBG, mutating the threonine in the P1 position of the reactive centre loop to an arginine by PCR-based site-directed mutagenesis. This CBG-T349R variant was found to have the same susceptibility to cleavage by thrombin as the CBG-antitrypsin_Pittsburgh_ chimera previously described (Figure 1). This confirms the feasibility of retargeting hormone binding globulins to deliver small molecules to new locations in the body by altering only a few residues on the reactive centre loop.

#### Targeting hyperplastic prostate tissue

Having demonstrated that the site specificity of hormone binding globulins can be altered with minor sequence changes, we went on to test an example with greater immediate relevance to cancer chemotherapy. Prostatic hyperplasias, both benign and malignant, are among the most common diseases in men, particularly in the developed world. Indeed, it was found that benign prostatic hyperplasia affects 70% of men over the age of seventy, and is the leading cause of lower urinary tract related morbidity [36]–[38]. Prostate cancer, a malignant form of prostatic hyperplasia, is the leading cause of cancer death in men living in the developed world. Although the etiological link between these two diseases is currently unclear, there is definitely a statistical association in their occurrences; importantly, they share common features, one of which is the overproduction of prostate-specific antigen (PSA) [38].

PSA belongs to the kallikrein-related peptidase (KLK) subgroup of serine proteases with chymotrypsin-like activity. PSA production typically increases with the enlargement of the prostate gland, regardless of the malignancy of the hyperplasia [39], making it one of the molecular markers used for the diagnosis of prostate hyperplasia, and an attractive target protease for an engineered CBG variant that is to deliver a therapeutic drug to hyperplastic prostate tissue. To engineer CBG's specificity, its reactive centre loop was mutated to mimic the substrate for PSA.

PSA has previously been reported to exhibit preferential binding for certain types of peptide sequences, and these small peptides typically contain a medium to large hydrophobic residue at the P1 position, a leucine at P2, a medium sized hydrophobic residue at P3, and a medium sized uncharged residue at P4 [40]. This corroborated an earlier report by Akiyama and coworkers, who used modified lysozyme and insulin as substrates for PSA, and found that the fastest rate of hydrolysis was obtained from the mutant bearing the SALLSSDI sequence in the P4 to P4′ position [41]. On the other hand, using a combination of phage display and iterative optimisation, Coombs and colleagues found that the P4 to P2′ sequence of SSYYSG was the best substrate for PSA [42].

**Figure 2:**
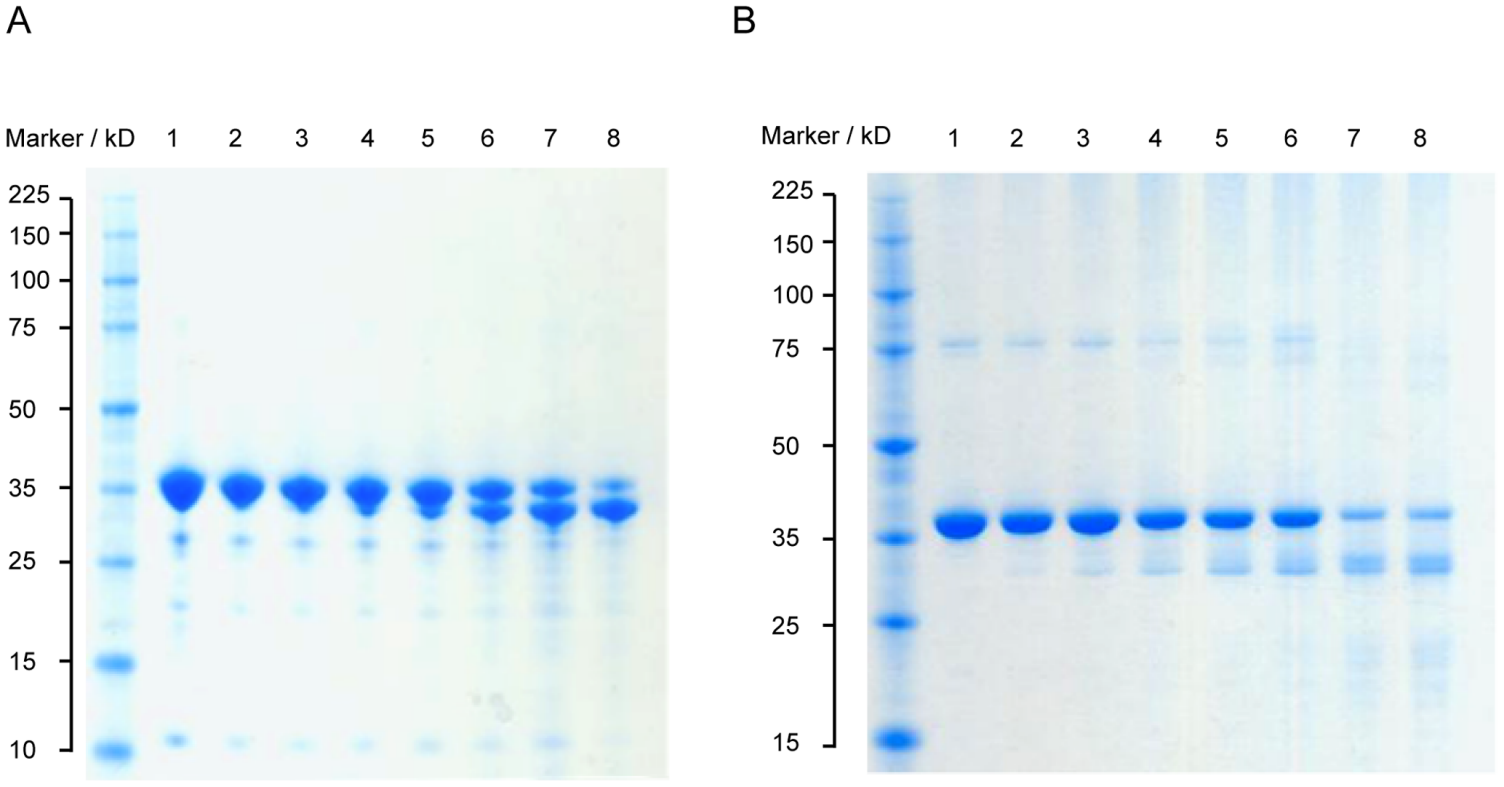
Prostate specific antigen (PSA) assay. Engineered CBG variants were incubated with PSA. In both gels, Lane1: uncleaved CBG; Lane 2: 2 hours incubation with PSA; Lane 3: 4 hours incubation with PSA; Lane 4: 8 hours incubation with PSA; Lane 5: 16 hours incubation with PSA; Lane 6: 24 hours incubation with PSA; Lane 7: 48 hours incubation with PSA; Lane 8: 72 hours incubation with PSA. (A) Of the two variants of PSA-sensitive CBG, the one with the reactive centre loop sequence EEGVDTAGSSYYSGNLTSKPII is cleaved the more rapidly. (B) CBG-EEGVDTAGSALLSSDITSKPII.

We mutated a segment of the wild type CBG's reactive centre loop from STGVTLNL to SALLSSDI and SSYYSGNL by site-directed mutagenesis, altering the presumed elastase cleavage site [43], which is located at residues P6-P5 in the canonical numbering for inhibitory serpins, to potential PSA cleavage sites on CBG. Unlike the CBG-T349R mutant, for which the protease cleavage site of the reactive centre loop was placed at the canonical P1 residue (in line with the crystal structure of the similar CBG-antitrypsin chimera that was previously solved by our group [19]) we had no *a priori* knowledge of the effect of inserting a PSA-specific sequence on the structure and biochemical behavior of CBG. The predicted PSA cleavage sites were therefore chosen to align with the main elastase cleavage site in wild type CBG, in order to keep the length of the reactive centre loop the same as the wild type in both the cleaved and intact forms. We then demonstrated that incubating the engineered CBG variants with PSA resulted in the cleavage of their respective reactive centre loops as shown in Figure 2, with the variant bearing the SSYYSGNL motif the better substrate for PSA. Inspection of this region in the structure of cleaved CBG shows that the change of the residues GV in native CBG to LL or particularly YY in the two PSA substrate mutants is likely to create steric strain on loop insertion. This would lead to a loss of stability and may render the cleaved mutant proteins more susceptible to further proteolysis, explaining the apparent reduction in total cleaved CBG (Figure 2).

As a control, we incubated wild type CBG with PSA for 72 hours and found no evidence of reactive centre loop cleavage (data not shown). As a consequence of the mutations, it is now possible for CBG to discharge its ligand where there are high levels of PSA, such as in hyperplastic prostate glands. Nevertheless, the current rate of reactive centre loop cleavage by PSA would need to be further optimised to a timescale suitable for drug delivery, as substantial cleavage was only seen after incubation for greater than 24 hours.

### Altering ligand specificity

CBG is particularly promising in the creation of a prostatic hyperplasia specific drug delivery system because of the properties of its ligand binding pocket. CBG's physiological ligands are steroid hormones, and it has a moderately high affinity for progesterone [44]. Since the 1980s, the synthetic progestin, megestrol acetate, has been used in the treatment of some forms of prostatic hyperplasia because it lowers the levels of testosterone, luteinising hormone and follicle-stimulating hormone, which are responsible for stimulating the unwanted proliferation of tissue in the prostate gland [45]–[49]. It also functions as an androgen receptor antagonist, starving prostate carcinomas of the testosterone and dihydrotestosterone required for their growth and maintenance [45], [48]. However, endocrinological treatment of prostate cancer is becoming less commonplace due to its reportedly limited efficacy in many cases [50]. Currently, the most common chemotherapeutic treatment for malignant metastatic prostate cancer is docetaxel, a mitotic poison [51]. In the next part of this proof-of-concept experiment, we sought to show that it is possible to alter the physicochemical characteristics of CBG's binding pocket sufficiently to bind a non-steroid compound.

#### Designing CBG to bind a thyroxine derivative

**Figure 3:**
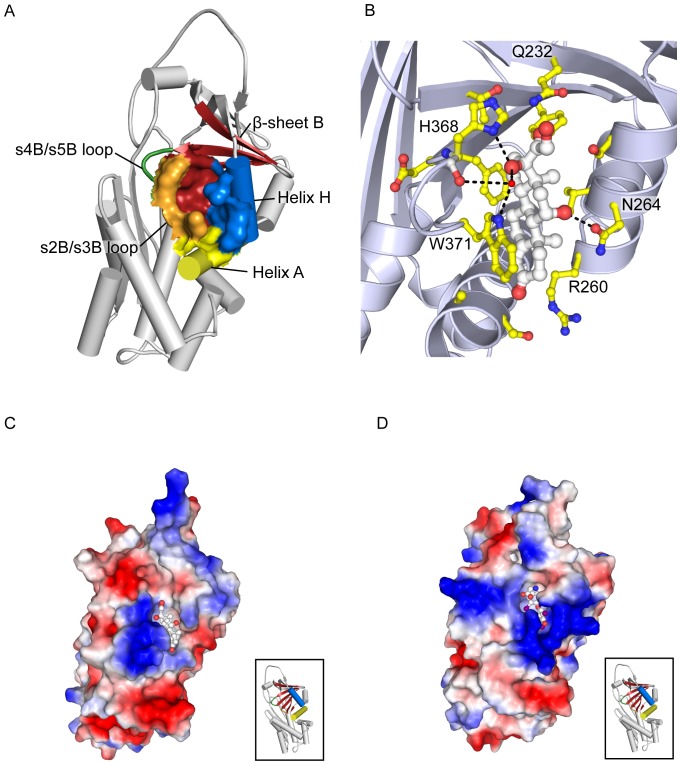
Ligand binding pocket of the hormone binding globulins. **(A)** The ligand binding pocket (solid surface, PDB entry 2VDY [19]) is formed by Helix A (yellow), Helix D (blue), β-sheet B (red), the s2B/s3B loop (orange) and the s4B/s5B loop (green). **(B)** R-state human CBG (PDB 2VDY [19], chain A) showing the interactions of pocket residues with cortisol. **(C)** Corticosteroid binding globulin (PDB 2VDY, chain A), and **(D)** Thyroxine binding globulin (PDB 2CEO [54], chain A). Electrostatic map: negative potential (red); positive potential (blue), uncharged/hydrophobic (white). **(C)** and **(D)** *(inset)*. Orientation represented in the electrostatic maps, with the key secondary structural elements coloured as in **(A)**.

CBG and its sister protein, TBG, have adapted to the function of hormone transport by developing a deep hydrophobic cleft in the region bounded by helix A, helix H, β-sheet B and the s2B/s3B and s4B/s5B loops, as shown in Figure 3A. This cleft serves as the binding pocket for both physiological and non-physiological ligands. As a carrier of glucocorticoid hormones, this pocket in CBG is lined primarily with uncharged amino acid residues, which complement the mainly hydrophobic surface of its steroid ligands. In fact, the main interaction between cortisol and CBG is thought to be the π-π stacking between the indole side chain of Trp 371 and the A, B and C cycloalkene rings of cortisol [18], [52]. A host of other hydrophobic interactions between the pocket residues and cortisol help contribute to the high affinity of binding, while other residues form a hydrogen bond network contributing to the selectivity of CBG for biologically-active C-21 steroids such as glucocorticoids [18], [19], [53]. These interactions are illustrated in Figure 3B.

TBG, on the other hand, binds thyroxine, which is a more polar molecule than cortisol. This is evident from the physical properties of the TBG binding pocket, which is considerably more polar and positively charged than that of CBG, as seen in Figures 3C and D. Arg 381 in TBG, like its analogous residue in CBG, Trp 371, appears to play an instrumental role in the protein-ligand interface. The positively-charged guanidium side chain of Arg 381 is believed to contribute to a cation-π interaction with thyroxine and its fluorescent derivatives [20], [54]. In this part of the study, we attempted to redesign the pocket of CBG to bind the thyroxine derivative, L-thyroxine-6-carboxyfluorescein.

To establish the importance of Trp 371 in CBG's interaction with cortisol, we first mutated it to alanine. The resultant CBG-Trp371Ala protein had no detectable affinity for cortisol when measured using the ForteBio Octet Red biolayer interferometry system. This confirmed previous inferences from biochemical [55] and structural [18], [19] studies about the crucial role of Trp 371 in the function of CBG. We then mutated Trp 371 to arginine, so that the binding site of CBG now begins to resemble that of TBG. Again, using biolayer interferometery, we found that CBG-Trp371Arg has no measurable affinity for cortisol. However, unlike wild type CBG, which showed no affinity for L-thyroxine-6-carboxyfluorescein, the CBG-Trp371Arg mutant was able to bind the thyroxine derivative, albeit at a lower affinity than TBG (CBG-Trp371Arg: K_d_ at 25°C  = 840 nM, TBG: K_d_ at 25°C  = 0.73 nM). This experiment has demonstrated the principle that the binding pocket of CBG can be redesigned by means of carefully chosen mutations to carry a non-physiological ligand. This same principle can be applied in the future to design CBG to carry other therapeutic compounds such as docetaxel to hyperplastic prostate tissue, or indeed any number of combinations of drugs and targets in the body.

### Increasing the amount of ligand released at the target

Next, we explored the fine-tuning of this drug-delivery vehicle to discharge a greater fraction of the bound ligand at the target site. We previously reported that recombinantly expressed wild type CBG undergoes a 2.6-fold decrease in affinity for cortisol upon cleavage of its reactive centre loop [21]. However, unlike cortisol, where a relatively small change in binding affinity to CBG is enough to perturb an existing equilibrium to result in a physiologically relevant change in free levels of the hormone, a much greater specificity of release is needed for therapeutic agents used for chemotherapy of solid tumours. In order to deliver a sufficiently high concentration of these drugs to the tumour by means of a protein carrier-based deliver system, a large proportion of the drug bound to the carrier needs to be released without affecting tissues outside the target.

For a protein carrier that binds the drug with a one-to-one stoichiometry, equation (1) describes the binding equilibrium. It can be seen from this equation that the concentration of free ligand is determined by the value of K_d_, and to achieve the dual aims of releasing biologically active amounts of therapeutic drug whilst avoiding off-target effects, the protein carrier would ideally bind its ligand with very high affinity (small K_d_) in the S-state, and experience a large decrease in binding affinity (large K_d_) upon reactive centre loop cleavage to release most of the bound compound at the target site in the R-state.

#### Replacing the P14 residue with a bulkier amino acid

**Figure 4:**
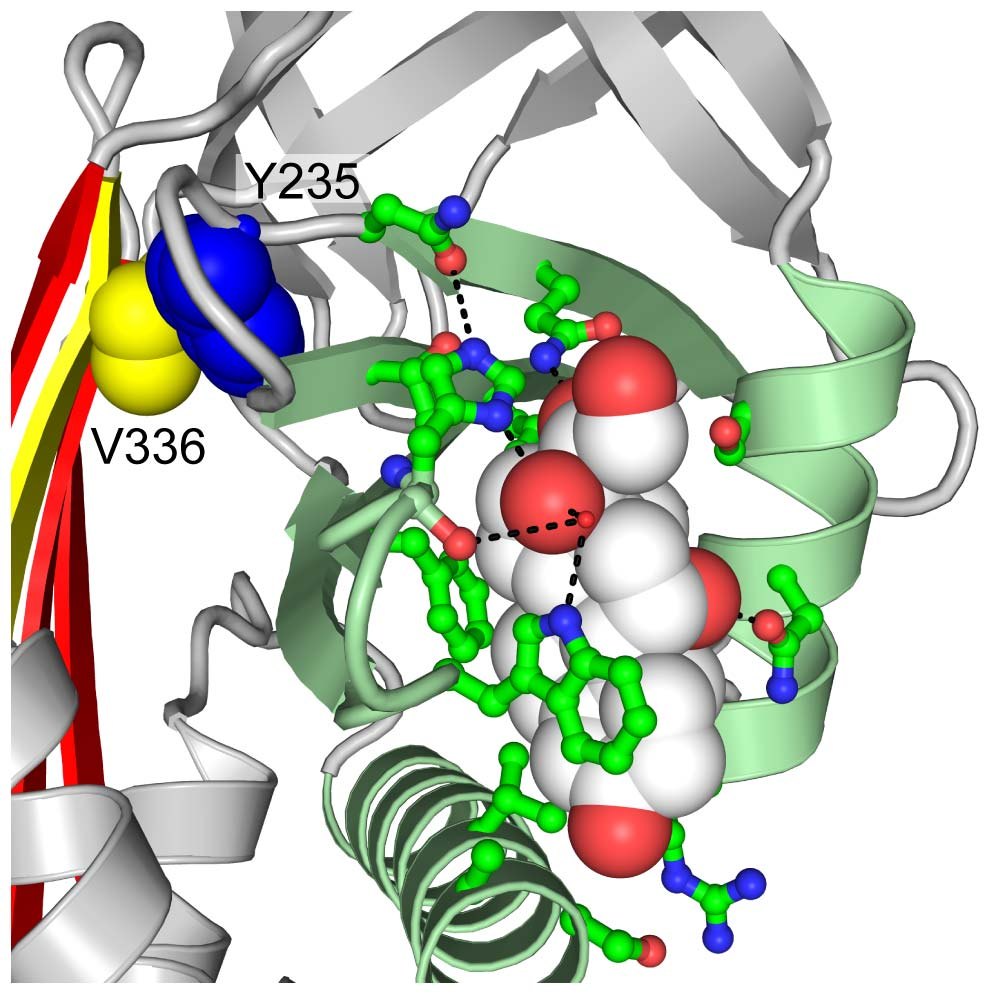
Reactive centre loop P14 residue. The P14 residue in CBG (PDB entry 2VDY [19]), Val 336, shown in yellow spheres, is believed to displace Tyr 235 (blue spheres) on the s2B/s3B loop when the reactive centre loop is inserted into the central β-sheet A (red), leading to conformational changes in the binding pocket (pale green). CBG's interaction with cortisol is provided by a network of side chains (bright green ball-and-stick representation), which can be perturbed by any slight movement in the pocket and its surrounding regions.

It has previously been reported for TBG that the P14 threonine on the reactive centre loop is a key component of the mechanism by which thyroxine is reversibly released by the S-state protein. The residue is believed to displace Tyr 241 on the neighbouring β-sheet B when the reactive centre loop of intact TBG is reversibly and partially inserted into β-sheet A, thus disrupting the hydrogen bond network that stabilises the steroid binding pocket [54], [56]. The same is believed to happen during the irreversible S-to-R conformational change, when the reactive centre loop is cleaved and inserted, this time irreversibly, into β-sheet A to form a novel fourth β-strand. Due to the structural homology of TBG and CBG, we inferred that the P14 valine in CBG might play a similarly important role (Figure 4).

By mutating this residue to amino acids with slightly bulkier side chains and similar chemical characteristics, we were able to amplify the effect of loop insertion on the adjacent hormone binding pocket without making it so sterically hindered as to prevent the process altogether. The two aliphatic natural amino acids chosen were leucine and isoleucine, with the mutations being made on a T349R background to allow cleavage by thrombin. These substitutions resulted in greater disruption to the ligand binding pocket upon reactive centre loop cleavage than valine in the wild type protein, and as a result, at physiological temperature, the binding affinity of CBG-V336L-T349R decreased by 8.2 times, while that of CBG-V336I-T349R decreased by 10 times following cleavage of its reactive centre loop (Table 2).

**Table 2:** Comparing the changes in cortisol binding affinity of CBG mutants.

CBG variant	Conformation	K_d_ (nM)	Fold change in K_d_
wild-type	S (uncleaved)	279±18	
	R (cleaved)	734±48	2.6
P1-Arg + V336I	S (uncleaved)	326±46	
	R (cleaved)	3420±440	10
P1-Arg + V336L	S (uncleaved)	295±44	
	R (cleaved)	2420±200	8.2
P1-Arg + S100C-V236C	S (uncleaved)	278±64	
	R (cleaved)	1800±49	6.5
P1-Arg + S100C-V236C-V336I	S (uncleaved)	297±40	
	R (cleaved)	2260±320	7.6
P1-Arg + S100C-V236C-V336L	S (uncleaved)	252±17	
	R (cleaved)	1600±320	6.4

Dissociation constants of various forms of CBG in the S- and R-states. Each pair of results for a given CBG variant represents the K_d_ of the intact S-state and the reactive centre loop-cleaved R-state (larger K_d_).

#### Covalently linking s2A/hD loop to binding pocket amplifies change in binding affinity

Based on our previous structures of CBG (PDB 2VDX, 2VDY [19]), we designed a mutant form of CBG that directly couples changes in helix D to the steroid binding pocket. Ser 100 on the s2A/hD loop and Val 236 on the s2B/3B loop of β-sheet B were mutated to cysteines so that under non-reducing conditions, they may be induced to form a disulphide bridge. This forms a direct covalent bond between the s2A/hD loop at the top of helix D and β-sheet B, which along with helices A and H make up the steroid binding pocket. We predicted that by covalently linking the s2A/hD loop to the binding pocket, any effect caused by the movement of the loop as a result of the S-to-R conformational change would be amplified.

The disulphide mutations were made on the T349R background to allow cleavage by thrombin. When binding assays were performed at 37°C in the presence of the reducing agent, TCEP, the change in binding affinity of CBG-S100C-V236C-T349R after reactive loop cleavage was very similar to that of wild-type CBG. However, in the absence of TCEP, cleavage of the reactive centre loop resulted in a 6.5-fold decrease in binding affinity for cortisol, as shown in Table 2.

**Figure 5:**
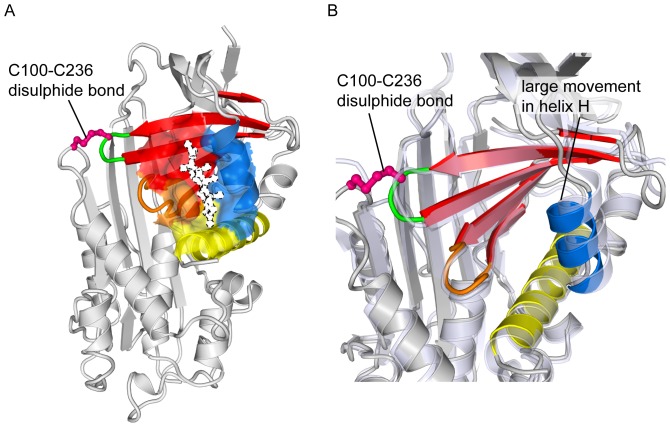
CBG-S100C-V236C-T349R structure. **(A)** Under non-reducing conditions, residues 100 and 236 form a disulphide bridge. Ligand binding pocket elements are identified by the same colour scheme as Figure 3. In this figure, cortisol from a previous structure (PDB 2VDY [19]) was superimposed on our unliganded structure (PDB 4C41, this work) to highlight the position of the pocket. It is represented as a white silhouette bound by broken lines. **(B)** When the disulphide bridge is formed, there is an obvious distortion of the steroid binding pocket, as shown here by superimposing the R-state structures of CBG (pale blue, transparent) and CBG-S100C-V236C-T349R (coloured as in **A**).

We went on to obtain the crystal structure of reactive centre loop-cleaved CBG-S100C-V236C-T349R, which revealed that the binding pocket was indeed perturbed by the presence of the disulphide bridge (Figure 5). Cleavage after Arg349 was supported by clear density for this residue, located at the bottom of sheet A after insertion of the RCL, and for Pro352, located more than 60Å away at the other end of the molecule, though density for Ser350 and Lys351 could not be interpreted unambiguously. The X-ray data also showed that the disulphide linkage has an occupancy of only about half, which suggests either that the disulphide bond is broken by radiation damage during data collection or, more likely, that even under non-reducing conditons, only about half of a given population of the CBG-S100C-V236C-T349R molecules have the desired disulphide bond at a given time. This could explain the relatively small effect of these mutations. Nonetheless, this amplified change in binding affinity is a definite step towards the rational optimisation of ligand release by CBG.

#### Combining mutations at Ser100Cys, Val236Cys and P14 residue of reactive centre loop

Having observed the augmented changes in cortisol binding affinities upon S-to-R conformational change either by covalently linking the s2A/hD loop with the steroid binding pocket, or by substituting the P14 valine with a bulkier residue, we tested if the effect of combining the two would be cumulative. It was found that the change in binding affinity of CBG-S100C-V236C-V336L-T349R was, in fact, diminished from the CBG-V336L-T349R mutant, and was only 6.4-fold. Similarly, the CBG-S100C-V236C-V336I-T349R mutant proved to have a mere 7.6-fold decrease in cortisol binding affinity following reactive centre loop cleavage (Table 2).

## Conclusions

In this study we have shown that, by means of targeted mutations, CBG can be engineered to bind and release compounds other than its physiological ligands, glucocorticoid hormones. In principle, this can be designed to occur at locations other than its physiological target, inflamed tissues. We hope that by proving the feasibility of this concept, the scope of future developments in targeted drug delivery can be expanded to include similar carrier proteins. Mutations made at and around the P1 residue of the reactive centre loop gave two CBG variants that had altered protease specificities, becoming susceptible to cleavage by either thrombin or prostate specific antigen. Since cleavage of the reactive centre loop triggers ligand release, there is a direct correlation between the protease specificity and CBG's site of action, and by targeting these engineered mutants to proteases that are localised to certain parts of the body, one can then direct the ligand to be released at very defined loci. Therapeutically, such a strategy could allow a lower total dose of the drug to be used while maintaining a higher local concentration of the compound at the desired site than current methods employed in chemotherapy. Moreover, since the drug is sequestered by the carrier protein until it is released at the target tissue, the risk of off-target effects would be minimised.

We also showed that, by carrying out site-directed mutagenesis of selected residues in the hormone binding pocket, CBG can be redesigned to carry a ligand of our choosing. By changing a binding pocket tryptophan normally involved in interaction with cortisol to an arginine, the ligand specificity of CBG was altered such that it now binds a thyroxine adduct, L-thyroxine-6-carboxyfluorescein, instead of cortisol. Using this same principle, one can potentially mutate the pocket to suit the chemistry of a wider variety of ligands. There are various computational tools to help in identification and redesign of residues involved in ligand binding so as to alter the ligand specificity more dramatically. Among them, the Rosetta protein modelling software suite has had some notable success in recent years [57], [58].

To increase the efficiency of ligand carriage and release, as well as to ensure that the amount of ligand released by the carrier whilst in circulation is negligible, the change in the dissociation constant, K_d_, upon reactive centre loop cleavage needs to be amplified. By mutating the P14 residue of the reactive centre loop to leucine and isoleucine, we have increased the change in binding affinity post-cleavage to about eight to ten-fold from the 2.6-fold difference seen in the recombinant wild type CBG. Covalently linking the s2A/hD loop to β-sheet B of the binding pocket with a disulphide bridge also resulted in a greater (6.6 times) loss in binding affinity, although the effects of the two alterations were not additive.

**Figure 6:**
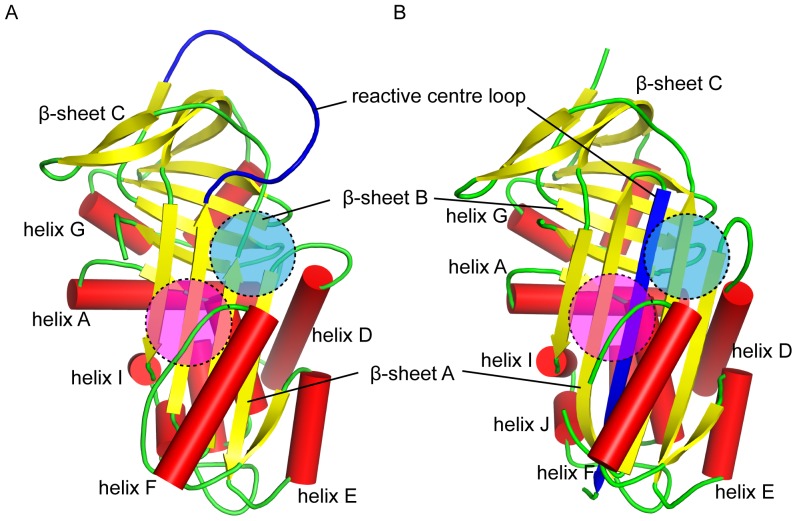
The conserved serpin fold. The canonical serpin fold comprises three β-sheets (yellow), eight to nine α-helices (red) and the reactive centre loop (blue). The proposed sites of the new ligand binding pocket are highlighted in blue (bounded by β-sheet A and helix D) and magenta (bounded by β-sheet A and helix F). (A) The reactive centre loop intact S-state of a typical serpin. (B) The reactive centre loop cleaved R-state of a serpin, showing the insertion of the loop into the middle of β-sheet A.

In the course of this project, we have come to appreciate that the release of hormone from the hormone-binding globulins is more subtle and modulated than was previously believed [21], and it has proven to be more difficult than anticipated to enhance the effect of cleavage. Perhaps if the ligand binding pocket were moved to a site where the conformation changes on cleavage are more pronounced, such as the region bounded by β-sheet A and helix D, or the region bounded by β-sheet A and helix F (Figure 6), the effects of S-to-R conformational change would have a significantly larger effect. Engineering a binding pocket ab initio, instead of making use of an existing site, would be a challenging task, but significant advances have been made by the Baker group in the development of Rosetta towards the computational design of proteins from scratch. In particular, they have demonstrated the rational design of novel proteins with small molecule binding sites in silico that have high affinities and specificities for their target ligands/substrates [57], [59], [60]. It is therefore reasonable to expect that the same technique can be used to build a high affinity binding site for a pre-specified ligand into an existing protein.

One possible risk associated with using a modified form of an endogenous protein as a drug carrier is the small but potentially dangerous possibility of raising an immune response against the mutant protein, which could affect the pharmacokinetics of the protein and/or result in hypersensitivity, as is observed in a small number of lysosomal storage disease patients receiving enzyme replacement therapy [61]. Just as worrying is the prospect of triggering an auto-immune response against endogenous CBG. However, we expect that by using a human protein as the starting scaffold, with only a small number of point mutations, most of which are not solvent exposed, the danger of developing an immune response against the drug carrier can be kept low. In the context of T cell-mediated responses, epitopes are typically defined by nonameric peptides [62], while B cell epitopes seem to involve about eight residues around an “epitope core” [63]. Moreover, seminal work on humanising mouse antibodies has demonstrated that retaining the mouse variable region, which makes up about 10% of an immunoglobulin, does not pose a problem with immunogenicity [64]. In general, it appears that it is often easier to destroy antigenicity than to create it, as a single point mutation has been known to destroy an epitope and result in immune evasion [65]. This combination of reasons suggests that in vivo administration of engineered CBG would not necessarily give rise to an immune response.

In summary, we have demonstrated in this study that by making targeted mutations to various structural features of CBG, we can, in principle, engineer the protein to carry a therapeutic drug and to release it upon cleavage of its reactive centre loop by site-specific serine proteases. Future work will need to strive towards optimising the efficiency of ligand release, and to test out other potential payload and protease specificities if this drug delivery system were to be successfully employed in vivo. Nonetheless, as a proof of principle, this study has opened the prospect of yet another means of targeted drug delivery to combat the persistent problem of off-target side effects in cancer chemotherapy.
